# Landslide Susceptibility Prediction Modeling Based on Remote Sensing and a Novel Deep Learning Algorithm of a Cascade-Parallel Recurrent Neural Network

**DOI:** 10.3390/s20061576

**Published:** 2020-03-12

**Authors:** Li Zhu, Lianghao Huang, Linyu Fan, Jinsong Huang, Faming Huang, Jiawu Chen, Zihe Zhang, Yuhao Wang

**Affiliations:** 1 Information Engineering School, Nanchang University, Nanchang 330031, China; lizhu@ncu.edu.cn (L.Z.); wanfy@email.ncu.edu.cn (L.H.); 416114417186@email.ncu.edu.cn (L.F.); 411014419075@email.ncu.edu.cn (Z.Z.); wangyuhao@ncu.edu.cn (Y.W.); 2 ARC Centre of Excellence for Geotechnical Science and Engineering, University of Newcastle, Newcastle, NSW 2308, Australia; jinsong.huang@newcastle.edu.au; 3 School of Civil Engineering and Architecture, Nanchang University, Nanchang 330031, China; jiawu_chen@email.ncu.edu.cn

**Keywords:** landslide susceptibility prediction, deep learning, cascade-parallel recurrent neural network, conditional random field, logistic regression, multilayer perceptron, decision tree, remote sensing, geographic information system

## Abstract

Landslide susceptibility prediction (LSP) modeling is an important and challenging problem. Landslide features are generally uncorrelated or nonlinearly correlated, resulting in limited LSP performance when leveraging conventional machine learning models. In this study, a deep-learning-based model using the long short-term memory (LSTM) recurrent neural network and conditional random field (CRF) in cascade-parallel form was proposed for making LSPs based on remote sensing (RS) images and a geographic information system (GIS). The RS images are the main data sources of landslide-related environmental factors, and a GIS is used to analyze, store, and display spatial big data. The cascade-parallel LSTM-CRF consists of frequency ratio values of environmental factors in the input layers, cascade-parallel LSTM for feature extraction in the hidden layers, and cascade-parallel full connection for classification and CRF for landslide/non-landslide state modeling in the output layers. The cascade-parallel form of LSTM can extract features from different layers and merge them into concrete features. The CRF is used to calculate the energy relationship between two grid points, and the extracted features are further smoothed and optimized. As a case study, the cascade-parallel LSTM-CRF was applied to Shicheng County of Jiangxi Province in China. A total of 2709 landslide grid cells were recorded and 2709 non-landslide grid cells were randomly selected from the study area. The results show that, compared with existing main traditional machine learning algorithms, such as multilayer perception, logistic regression, and decision tree, the proposed cascade-parallel LSTM-CRF had a higher landslide prediction rate (positive predictive rate: 72.44%, negative predictive rate: 80%, total predictive rate: 75.67%). In conclusion, the proposed cascade-parallel LSTM-CRF is a novel data-driven deep learning model that overcomes the limitations of traditional machine learning algorithms and achieves promising results for making LSPs.

## 1. Introduction

Landslides are one of the most common geological disasters worldwide and cause considerable damage to public infrastructure and human life every year [1,2]. The predictive modeling of landslide occurrence is one of the main challenges in geological hazard research. A landslide susceptibility map (LSM) is an effective visualization technology for the localization of a landslide region and sustainable land management [3,4,5]. Moreover, the landslide susceptibility model based on geological environmental conditions can supply the government with an important theoretical basis for land resource planning and disaster prevention and reduction.

The process of landslide susceptibility prediction (LSP) modeling primarily includes a catalog of landslides, environmental factors extraction, model architecture construction, model training, landslide susceptibility mapping, and model evaluation [6,7]. The catalog of landslides (landslide area, boundary, locations) are measured using global positioning systems and put into a geographic information system (GIS) for landslide storage and management [8,9]. The environmental factors are extracted from the remote sensing (RS) images, such as Landsat8 TM image, digital elevation model (DEM), aerial imagery, and LiDAR, based on the GIS spatial analysis, including terrain analysis, hydrological analysis, and map algebra [10]. As a whole, the LSP modeling is built on the platform of GIS because of the spatial big data analysis, storage, mapping, and management abilities [11]. 

Importantly, based on the above obtained spatial data sources, the input data, network architecture, parameter settings, and optimization algorithm of the model all affect the accuracy of landslide predictions. In recent decades, researchers have developed various predictive models combined with the GIS, which includes heuristic models and statistical models [12], e.g., information value model [13], logistic regression [14,15], entropy index [16], certainty factor [17,18], analytic hierarchy process [19,20], etc. However, these models have certain limitations in LSP applications. For example, models often require feature data to be subject to a certain statistical distribution or an independent and identically distributed assumption, but environmental factors often fail to meet these prior knowledge requirements. In recent years, machine learning methods have been widely used in landslide susceptibility modeling and have achieved remarkable results because of their high prediction accuracy and absence of a prior knowledge requirement. As such, this approach produces a higher prediction accuracy, can more precisely identify the nonlinear relationship between input and output variables, and retains more characteristic information from the original data [21,22,23,24]. This approach includes multiple adaptive regression splines [25,26], fuzzy logic [27,28], artificial neural network [15,29], multilayer perceptron [30], decision tree [31,32,33], random forest [34,35,36], support vector machine [37,38,39], rule-based approach [40], and multi-criteria evaluation techniques [41], among others.

Selected disadvantages of traditional machine learning models occur in the application of LSP: (1) the models generally require a large amount of prior knowledge and assumptions; (2) the networks are not sufficiently deep to fully extract the underlying landslide features; (3) the networks are not sufficiently wide to consider the correlations between sub-regions; and (4) the models encounter problems, such as over-fitting, time-consuming computation, ease of falling into local optima, and sensitivity to missing data, which affect the accuracy of prediction. 

Deep learning is an emerging multilayer neural network learning algorithm that can overcome the shortcomings of traditional machine learning models to a certain extent. Compared with traditional machine learning methods, deep-learning-based models are capable of extracting inherent and deep features. Deep learning methods are data-driven without requiring additional prior knowledge or assumptions [42,43,44], and can effectively identify useful information among miscellaneous data and obtain the optimal parameters for constructing models in the process of model training. At the same time, the impact of over-fitting on model prediction accuracy can be eliminated by using a considerable number of iterations. Moreover, as advanced data mining models, deep learning algorithms have been widely used in various fields, such as image recognition [44], face recognition [45], medical artificial intelligence [46], natural hazards mapping [47], etc. Due to their strong capability of feature extraction, it is necessary to apply deep learning methods to predict the landslide susceptibility in the study area [48]. The current basic models of deep learning include the deep neural network, convolutional neural network, recurrent neural network, and a series of new structures, such as the long short-term memory (LSTM) [49] structure and residual network (ResNet) [50], which are state-of-the-art models with a high performance across numerous applications and are valuable in the theoretical study of deep learning, among others. In addition, many methods, such as the rectified linear unit activation function, have been developed to solve the problems of gradient disappearance and over-fitting in traditional networks. Moreover, model calibration is necessary for landslide susceptibility prediction. Venables and Ripley [51] present a guide to using S environments to perform statistical analyses, practical problems, and real data sets. Common calibration methods for landslide succeptibility (LS) assessment includes (i) linear discriminant analysis (LDA) [51,52,53], (ii) quadratic discriminant analysis (QDA) [53], (iii) logistic regression (LR) [54], and (iv) neural network (NN) modeling [53,55]. A comprehensive calibration for neural-network-based LSP modeling aims to improve the output characteristic of all nodes, resulting in a computationally intensive problem.

In summary, landslide susceptibility prediction plays a significant role in resisting landslides and reducing disasters. It is also a challenging problem because landslide features are generally uncorrelated or nonlinearly correlated, resulting in limited LSP performance when leveraging traditional machine learning models. To overcome the limitations where traditional machine learning algorithms require substantial prior knowledge and achieve promising results regarding LSPs, a novel data-driven deep learning model using the LSTM recurrent neural network and conditional random field (CRF) in cascade-parallel form was proposed for LSP based on the remote sensing (RS) images and a geographic information system (GIS). In the proposed model, the cascade-parallel LSTM can extract and merge features from different layers without prior knowledge, and CRF is used to further smooth and optimize those extracted features. Meanwhile, a dropout strategy is adopted to prevent the problem of over-fitting in traditional neural networks. 

The study and comparison of machine learning models based on deep learning algorithms and other traditional models for LSP is of great significance. Taking Shicheng County in China as a case study, this study proposed a recurrent neural network, namely a cascade-parallel LSTM model, to predict landslide susceptibility in the study area. Considering the influence of the relationship between adjacent grids on landslide susceptibility, the conditional random field (CRF) was further introduced to optimize the models. Furthermore, logistic regression (LR), multilayer perceptron (MLP), and C5.0 decision tree (C5.0 DT) methods were selected for analysis and comparison.

## 2. Materials and Methods

### 2.1. Materials

#### 2.1.1. Introduction to Shicheng County

**Figure 1:**
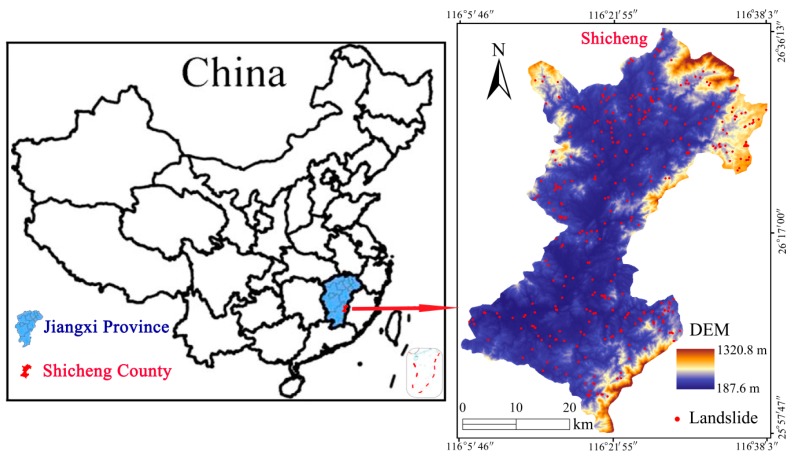
Location map in the study area. DEM: Digital elevation model.

Shicheng County is located in the northeast portion of Ganzhou City, Jiangxi Province, with a longitude ranging from 116°05′46″–116°38′03″ N, a latitude ranging from 25°57′47″–26°36′13″ E and a total area of 1581.53 km^2^, as illustrated in Figure 1. Shicheng County is a typical southeast low mountain and hilly region, the topography of which is enveloped by many mountains in the northeast, rolling hills in the southwest, and flat terrain in the middle. Moreover, this region resides in a subtropical humid monsoon climate zone with ample sunshine, an annual mean temperature of 18.1 ℃, and abundant rainfall, where the annual precipitation is 1919.6 mm. Furthermore, Shicheng basin formation in this area is attributed to the influence of tectogenesis, and the Guifeng Group and the Late Cretaceous of the Ganzhou Group are its main outcropping strata. The Guifeng Group is the prime landscape formation of the Danxia landform, which is divided into the Hekou formation with cracked landforms in its red sandstone, and the Tangbian and Lianhe formations, from bottom to top. The Anyuan-Yingtan fault zone, the Caledonian Indosinian-Huali granite, and the Cretaceous basin, which are distributed in the form of beads, are located on the west side of the basin. The Heyuan-Shaowu fault zone, which has obvious control of the formation and distribution of the Meso Cenozoic basin, is located on the east side.

#### 2.1.2. Landslide Distribution Map

The catalog of the landslide distribution map is the key to the LSP, where the accurate landslide locations and detailed geological information are primarily reflected. The landslide locations are acquired based on historical landslide disaster reports, field investigations, and interviews with residents conducted by the Land and Resources Department of Jiangxi Province. A total of 369 landslides have been recorded in Shicheng County from 1970 to 2012 (Figure 1). The main features of these landslides can be described as small-scale, high-frequency, with a wide distribution.

The total area covered by the landslides in Shicheng County is about 2.44 × 10^6^ m^2^ with the smallest area being 1.0 × 10^3^ m^2^ and the largest area being 1.6 × 10^4^ m^2^. The landslide masses are generally composed of quaternary silty clay intercalated with crushed stones, and the depth of these sliding masses ranges from 2 m to 8 m. In addition, these landslides can be regarded as shallow soil landslides with a movement type of clay/silt slide [56]. Finally, the landslides in Shicheng County are mainly triggered by the seasonal heavy rainfall and unreasonable constructive activities (such as slope toe cutting and road network construction)

### 2.2. Environmental Factors Extraction from RS and GIS

A total of 14 environmental factors were extracted from RS and the GIS, including topographic, land cover, hydrological, and lithology factors.

#### 2.2.1. Terrain Analysis Using DEM Data

The topographic factors (including elevation, slope, aspect, profile curvature, plan curvature, and relief amplitude) and hydrological factors (distances to rivers and topographic wetness index (TWI)) were extracted from the DEM with a 30-m resolution (http://gdem.ersdac.jspacesystems.or.jp). The profile curvature represents the vertical plane parallel to the slope direction; it is calculated as the slope of the slope using the terrain analysis tool in ARCGIS 10.2, ESRI, USA. Meanwhile, the plan curvature represents the various features of the concave terrain from the horizontal direction and is calculated as the slope of the aspect [57] in ARCGIS software. Meanwhile, the relief amplitude, which represents the slope surface relief characteristics of Shicheng County, was calculated using the maximum height difference method in ARCGIS software [58]. 

#### 2.2.2. Land Cover Factors Acquisition from Google Earth and Landsat TM8 Images 

The environmental factors of the total surface radiation and population density were downloaded from Google Earth 7.1.8.3036 (32-bit). The total surface radiation, defined as the sum of direct and diffuse solar radiation received on the horizontal surface, affects the hydrological environments and land cover types of the slope [59]. Population density, which represents the population in a certain area, mainly plays an important role in human construction activities [60].

The Landsat TM8 image taken on 5 October 2013, path/row 121/42 with a 30-m resolution (http://ids.ceode.ac.cn/index.aspx) was used to extract the land cover factors, which are expressed using the normalized difference vegetation index (*NDVI*), the modified normalized difference water index (*MNDWI*), and the normalized difference build-up index (*NDBI*) [61]. *NDVI* mainly represents the vegetation growth and cover rates of the study area (Equation (1)). *MNDWI* represents the surface water distribution features, as shown in Equation (2). In addition, the *NDBI* reflects the building distribution features on the surface of the landslide, as shown in Equation (3). The P(B3), P(B4), P(B5), and P(B6) represent the visible green band, visible red band, near-infrared band, and middle infrared band, respectively, of the Landsat 8 TM image.

(1) $$NDVI=\frac{P\left(B5\right)-P\left(B4\right)}{P\left(B5\right)+P\left(B4\right)}$$

(2) $$NDBI=\frac{P\left(B6\right)-P\left(B5\right)}{P\left(B6\right)+P\left(B5\right)}$$

(3) $$MNDWI=\frac{P\left(B3\right)-P\left(B6\right)}{P\left(B3\right)+P\left(B6\right)}$$

#### 2.2.3. Analysis of Hydrological Factors

The effects of hydrological factors on landslides are reflected through the topographic wetness index (*TWI*) and the distances to rivers. *TWI* represents the effects of topography and soil moisture content on the probability of landslide occurrence. The distances to rivers represents the distance of grid cells to the rivers and drainages in the area, suggesting the effects of surface water on landslides. The distances to rivers can be calculated based on the multi-ring buffering method in ARCGIS software.

The river networks of the study area are extracted through the hydrological analysis tool [62]. First of all, the DEM data was filled by the fill tool of the hydrological analysis tool to handle the defects of the original data. Second, the flow direction was calculated based on the filled DEM data, then the flow accumulation of each grid cell was calculated based on the flow direction and the filled DEM. Third, the flow accumulation threshold was set to 5000 according to the trial-and-error method [63], and the grid cells with a flow accumulation threshold greater than 5000 could form the river networks. Finally, a river network with a linear vector format could be obtained through the grid turn line tool in ARCGIS software. 

*TWI*, representing the impacts of topographic factors on the soil moisture content along the runoff areas, has been widely used to describe the hydrological influences on landslide occurrences. *TWI* can be expressed as given in Equation (4), where $A_{s}$ refers to the upstream catchment area and β represents the slope angle of a certain grid cell:

(4) $$TWI=\ln\left(A_{s}/\tan\beta \right)$$

#### 2.2.4. Analysis of the Lithology Factor

Lithology is represented by the rock types, which are the material basis of landslide development, with a great influence on the shear strength and permeability of the rocks and soils in the landslide mass. In this study, a rock type map was provided by the Land and Resources Department of Jiangxi Province and was mapped through the spatial analysis tool of ARCGIS software. The rock types in Shicheng County are mainly defined as metamorphic, carbonate, and clastic rocks.

### 2.3. Frequency Ratio (FR) Method

The application of the *FR* method is usually based on the assumption that future landslide events will occur in the geological environment of the past and present landslides [48,64]. Moreover, *FR* quantifies the relationship between the observed landslides and each environmental factor and is defined as the ratio of the proportion of landslide grid numbers in each subclass factor to the proportion of study area grid numbers in each subclass factor. The higher the *FR*, the higher the probability that a landslide will occur in the subcategory of the corresponding factors:

(5) $$FR_{ja}=\frac{S_{ja}^{\prime }/S}{M_{ja}^{\prime }/M}$$

where $FR_{ja}$ is the frequency ratio of the ath subcategory in the jth environmental factor, $S_{ja}{}^{\prime }$ is the number of landslide grids of the ath subcategory in the jth environmental factor, S is the total number of landslide grids, $M_{ja}^{\prime }$ is the number of study grids of the ath subcategory in the jth environmental factor, and M is the number of total grids in the study area.

### 2.4. Modeling Processes of Cascade-Parallel LSTM-CRF

The proposed cascade-parallel LSTM-CRF model for LSP consists of five steps:

(1) A large landslide inventory and related environmental factors are collected from a global positioning system, RS, and GIS technologies.
(2) The frequency ratio values (*FR*s) of those environmental factors are calculated and labeled, and then are used as input variables of the machine learning models.
(3) The landslide and non-landslide grid cells are used as the output variables of these models.
(4) The landslide susceptibility models are built based on the cascade-parallel LSTM-CRF, as well as the other models for comparisons.
(5) The LSMs of Shicheng County and the model accuracy evaluation are performed.

### 2.5. Theory of Cascade-Parallel LSTM-CRF

**Figure 2:**
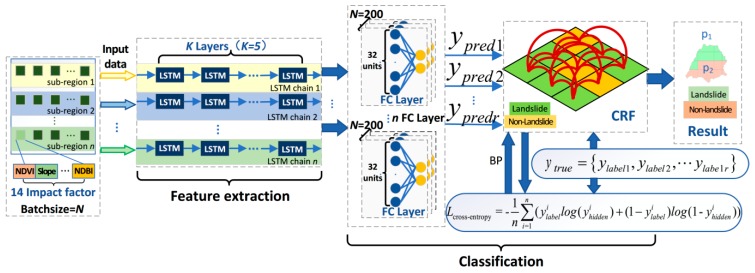
Algorithm flowchart. BP: Back propagation, CRF: Conditional random field, FC: Full connection, LSTM: Long short-term memory.

In this study, the cascade-parallel LSTM and CRF were proposed for LSP, as shown Figure 2. The model consists of sub-region feature modeling, fully connected layer classification, and implicit state modeling. The proposed model has a higher LSP performance and overcomes the limitations in terms of achieving a wider range of landslide data prediction. With the stacked structure, it can extract more comprehensive and accurate landslide features, which facilitates classification. Furthermore, CRF can optimize the extracted features and smooth the predicted results of mutations.

#### 2.5.1. Sub-Region Feature Modeling

The modeling area is first divided into consecutive sub-regions $S^{e}$, (e=1, 2,…,⌈M/N⌉), where M is the number of grids in the whole area, N is the defined number of grids in each sub-region, and ⌈•⌉ denotes the ceiling operation (i.e., x is rounded up to the nearest integer). The *FR* vectors $x^{e}$ of the J environmental factors in the eth sub-region are used as the raw input data of the eth cascade LSTM. As shown in Figure 2, the batch size is equal to the number of grids in each sub-region. The batch gradient descent (BGD) method is used in each sub-region. In light of the complicated characteristics of landslide factors, this section uses cascade LSTM [65] to extract the corresponding features of landslides, which shows superior performance over the single LSTM.

**Figure 3:**
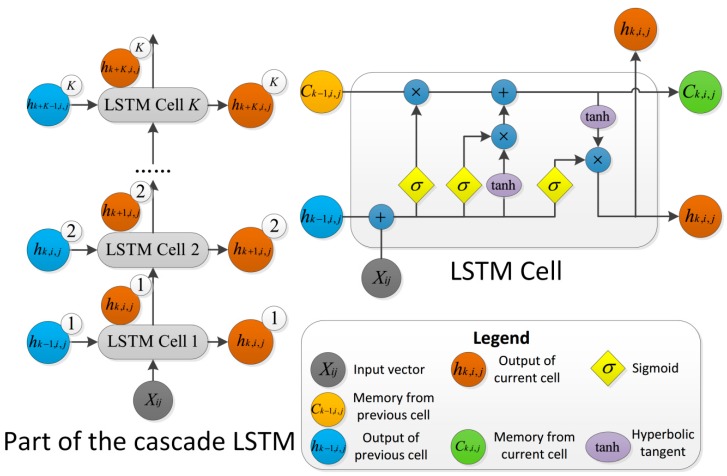
LSTM structure diagram.

By leveraging the cascade of K LSTM cells and fully connected feed-forward units, the deep features and space patterns of the input *FR* vectors $x^{e}$ are extracted for landslide/non-landslide classification. The LSTM cell is shown in Figure 3.

Due to the high coupling and sophisticated nonlinear correlations among landslide environmental factors, landslide environmental factors cannot be used as features of prediction. Therefore, it is necessary to establish a cascade deep network structure that extracts effective features of different weights from different layers by decoupling the complex relationships through multiple implicit layers. The input layer of the cascade-parallel LSTM-CRF is the real matrix $R^{i\times j}$ of the landslide environment factors, where the row vector {i |1≤i≤M,i∈Z} is the number of grids and M is the total number of grids, the column vector {j |1≤j≤J,j∈Z} is the number of landslide environment factors and J is the total number of landslide environment factors, and $R^{M\times J}$ passes through an implicit layer of K LSTM cells cascaded in series. The LSTM unit is shown in Figure 3.

When the kth LSTM cell is processing the data of the jth environmental factor of the ith grid point using forward propagation, the update formulas for the forget gate layer ($f_{k,i,j}$), the input gate layer ($i_{k,i,j}$), and the output gate layer ($o_{k,i,j}$) are written as follows:

(6) $$f_{k,i,j}=\sigma \left(W_{f}\cdot \left[h_{k-1},x_{ij}\right]+b_{f}\right)$$

(7) $$i_{k,i,j}=\sigma \left(W_{i}\cdot \left[h_{k-1},x_{ij}\right]+b_{i}\right)$$

(8) $$o_{k,i,j}=\sigma \left(W_{o}\cdot \left[h_{k-1},x_{ij}\right]+b_{o}\right)$$

where “σ” represents the sigmoid function used as the activation function; $W_{f}$, $W_{i}$, and $W_{o}$ are the weight matrices of each gate of the LSTM cell; $h_{k-1}$ and $x_{ij}$ represent the hidden state from the previous unit and the jth environmental factor of the ith grid point, respectively; $b_{f}$, $b_{i}$, and $b_{o}$ are the corresponding bias terms; and $f_{k,i,j}$ outputs a number between 0 and 1. The internal data calculation process of LSTM is as follows:

(9) $${\tilde{C}}_{k,i,j}=\tanh\left(W_{c}\cdot \left[h_{k-1},x_{ij}\right]+b_{C}\right)$$

(10) $$C_{k,i,j}=f_{k,i,j}*C_{k-1}+i_{k,i,j}*{\tilde{C}}_{k,i,j}$$

(11) $$h_{k,i,j}=o_{k,i,j}*\tanh(C_{k,i,j})$$

where $C_{k,i,j}$ is the current cell state, ${\tilde{C}}_{k,i,j}$ is the candidate state generated by the current input gate layer based on the previous hidden layer, and $h_{k,i,j}$ is the hidden layer state. First, the input gate layer generates the candidate state value ${\tilde{C}}_{k,i,j}$. Second, the forget gate layer decides which information is discarded (when $f_{k,i,j}$ = 1, it means that the previous cell state is completely retained; when $f_{k,i,j}$ = 0, it means that the previous cell state is completely discarded). The value of the new candidate state is added to the input gate layer to create an update of the cell state. Finally, the output gate layer outputs $C_{k,i,j}$ and the hidden variable $h_{k,i,j}$ based on the current cell state. During the process of forward propagation, the formula for the change from the input of raw data to the output from cascade LSTM is as follows:

(12) $$y_{out}=\prod_{k=1}^{K}\sum_{i=1}^{M}\sum_{j=1}^{J}o_{k,i,j}*\tanh\left(f_{k,i,j}*C_{k-1}+i_{k,i,j}*C_{k,i,j}\right)$$

where M, J and K represent the total number of grid points, the environmental factors of the single grid point, and the LSTM cells, respectively. The internal weights of the LSTM cells and bias parameters are updated using backpropagation. When the kth LSTM cell is processing the ith grid, the error of an output cell is as follows:

(13) $$\delta_{k}^{}\left(i\right)=\frac{\partial L_{cross-entropy}}{\partial x_{i}}$$

The error of the entire output gate layer can be readily computed based on the error of the output cell as follows:

(14) $$\delta^{o}\left(i\right)=\sigma^{\prime }\left(o\left(i\right)\right)\left(\sum_{v=1}^{K}\tanh\left(h_{v}\left(i\right)\right)\sum_{k}^{}W_{c}^{k}\delta_{k}\left(i\right)\right)$$

The three gate layers of the LSTM act as three switches, which have the function of the selective forgetting of old cell states, selective logging of input information, and selective determination of which portion of the cell state is output. The three gates of LSTM [66] control the state of the cells, which are similar to the conveyor belt, making one-dimensional data transmit throughout the chain smoothly. Hence, LSTM is ideal for processing 1D (one-dimensional) landslide raw data. In this study, the algorithm can fuse the features of each layer of the LSTM through the data stream connection between layers, which extracts more abundant features than the single layer of LSTM.

To prevent the model from over-fitting during training, a dropout process was adopted in the LSTM cells. The specific process is described as follows. First, in a batch of training samples, a portion of neurons in the LSTM cells are randomly deleted, and the remaining neurons are fed to the next layer. Second, after obtaining this batch of training samples, the deleted neurons are restored, and certain neurons in the LSTM cells are randomly removed once again. The corresponding parameters are updated via the Adam method, which is performed on the un-removed neurons. This process is repeated. The dropout in this study was set to 0.2, which remarkably reduced the probability of over-fitting. This approach is beneficial to the subsequent independent and non-redundant optimal feature extraction. For the entire area, ⌈M/N⌉ sub-regions are predicted with parallel implementation. The cascading LSTMs are applied to each sub-region.

#### 2.5.2. Fully Connected Layer Classification 

LSP is a two-category task. Therefore, a classification layer is required in the model to predict whether the grid has a landslide. The K cascade LSTM extracts the 32-dimensional landslide features of the landslide factors for each grid, and all features form a fully connected layer. A classification layer formed by two neurons follows the fully connected layer. This process maps the 32-dimensional feature space to the 2-dimensional space through a sigmoid function, i.e., the landslide/non-landslide space. Additionally, the nonlinear sigmoid function maps the real features in (−∞,+∞) to the landslide probability values in [0,1], producing a landslide prediction.

#### 2.5.3. Hidden State Modeling 

The extracted features are observed states, and the landslide/non-landslide is the hidden state. With the fully connected output as the conditional random field input, the extracted features are further optimized and classified. For the LSP task (or general structured prediction), it is beneficial to consider the correlations between labels in neighborhoods, and it is reasonable to assume that the labels in each input sub-region are correlated.

The landslide factor is the observed state, and the landslide/non-landslide is the implied state. The prediction results obtained by the fully connected layer are all from the observed state, without consideration of the spatial correlation between the hidden states. For example, if the grids are spatially related and other raster predictions in a certain neighborhood of a certain grid are landslides, then the grid is more likely to contain a landslide. For example, based on the assumption that a spatial correlation exists between grids, if other grids in a certain neighborhood of a grid are predicted to be landslides, then this grid is more likely to contain a landslide. In this study, according to the order characteristics of landslide data, a CRF [67,68] is introduced after the fully connected layer for hidden state modeling to produce a landslide/non-landslide prediction sequence $y_{hidden}=\{y_{hidden}^{1},y_{hidden}^{2},\cdots ,y_{hidden}^{n}\}$ and make more accurate predictions. At the same time, CRF can smooth out the sudden changes in the prediction results and correct the unreasonable prediction. Assuming that the fully connected layer output $y_{hidden}=\{y_{hidden}^{1},y_{hidden}^{2},\cdots ,y_{hidden}^{n}\}$ and the true label sequence during the training process phase $y_{label}=\{y_{label}^{1},y_{label}^{2},\cdots ,y_{label}^{n}\}$ satisfy the Markov property, then the conditional probability $p\left(y_{lable}|y_{pred}\right)$ is calculated as follows:

(15) $$p\left(y_{lable}|y_{pred}\right)=\frac{1}{Z\left(x\right)}\exp\left(\sum_{i,k}\lambda_{k}t_{k}\left(y_{label}^{i-1},y_{label}^{i},y_{pred},i\right)+\sum_{i,l}\mu_{l}s_{l}\left(y_{label}^{i},y_{pred},i\right)\right)$$

where $Z\left(y_{pred}\right)=\sum_{y}\exp\left\{\sum_{i=1}^{n}w_{i}f_{i}\left(y_{lable},y_{pred}\right)\right\}$ denotes the normalized factor, $t_{k}$ is the transfer characteristic function,$s_{l}$ is the transfer characteristic function, and $\lambda_{k}$ and $\mu_{l}$ are the corresponding weight coefficients. In this study, the maximum likelihood estimation method was used to optimize the CRF. The likelihood function is given as follows:

(16) $$L\left(W\right)=\sum_{i}\log p\left(y_{label}^{i}|y_{label}^{i}\right)$$

The goal of optimization is to obtain the maximum conditional probability of the above formula. This study used the Viterbi algorithm to optimize the CRF calculation process. The final prediction result $y_{hidden}$ is obtained from the output $y_{pred}$ of the fully connected layer and the conditional probability $p\left(y_{lable}|y_{pred}\right)$ calculated using the CRF. 

#### 2.5.4. Loss Function 

A cross entropy is introduced as the loss function of the entire model to achieve optimization and convergence. The loss function based on cross entropy is written as Equation (17). The reasons for choosing cross entropy in this study are listed as follows: (1) Because the difference in the original data of the landslide factor is relatively small, the logarithmic function in the cross entropy is used to expand the gap between the data, thereby reducing the numerical calculation error. (2) Because the influences of different influence factors on the landslide are different, the cross entropy is introduced to ensure that each impact factor has a different weight. The model can find the direction of the fastest gradient descent and accelerate the convergence of the model. (3) Cross entropy makes the probability of optimization more accurate, i.e., the probability of a landslide is closer to 1, and the probability of a non-landslide is closer to zero.

(17) $$L_{\mathrm{cross}-\mathrm{entropy}}=-\frac{1}{n}\sum_{i=1}^{n}\left(y_{label}^{i}log(y_{hidden}^{i}\right)+(1-y_{label}^{i})log(1-y_{hidden}^{i}))$$

## 3. Results

### 3.1. Landslide-Related Spatial Dataset

#### 3.1.1. Landslide-Related Environmental Factors

**Figure 4:**
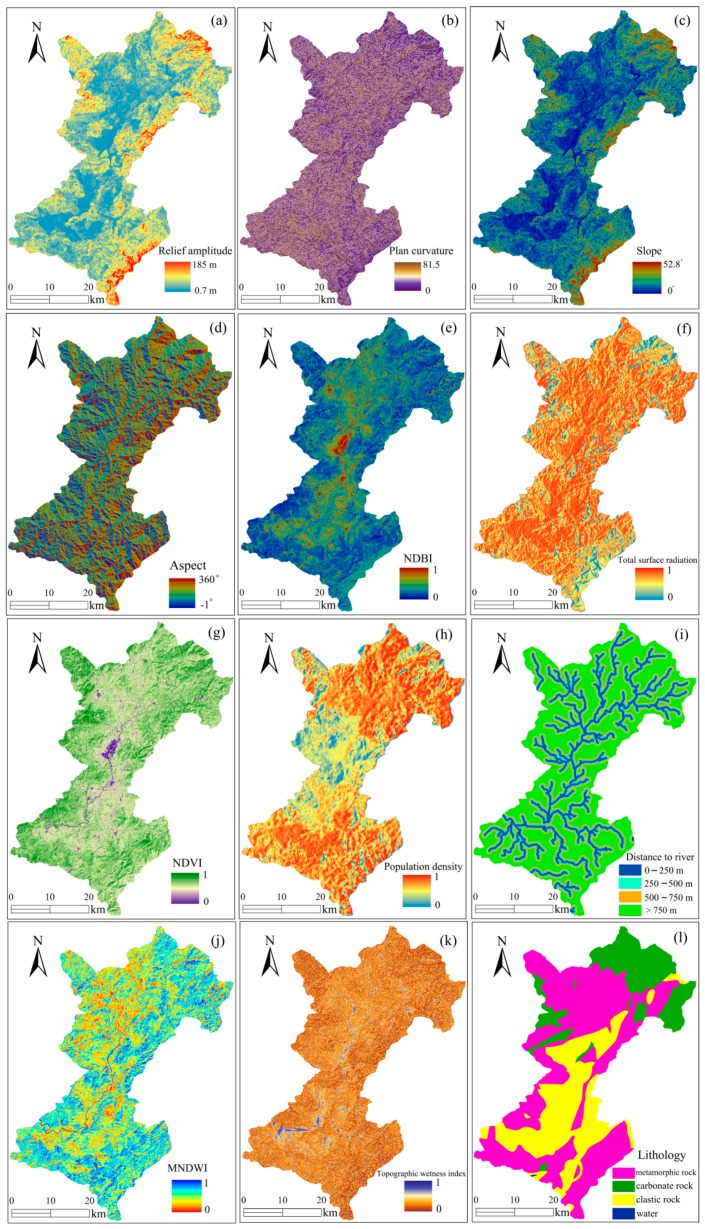
Landslide environmental factors (elevation and profile curvature are not presented): (**a**) Relief amplitude, (**b**) Plan curvature, (**c**) Slope, (**d**) Aspect, (**e**) NDBI, (**f**) Total surface radiation, (**g**) NDVI, (**h**) Population density, (**i**) Distance to river, (**j**) MNDWI, (**k**) Topographic wetness index, (**l**) Lithology.

In the existing study, no agreement was found regarding the causes of landslide events due to the complexity of the geological environment and the sensitivity of landslide evolution [69]. However, based on studies between landslide and environmental factors conducted by researchers in different regions, the environmental factors consist of five main categories: topography and geomorphology, hydrologic environment, basic geology, land cover, and human activities [70]. Therefore, 14 environmental factors were selected as input variables for landslide prediction in this study: elevation, slope, aspect, plan curvature, profile curvature, relief amplitude, *NDBI*, total surface radiation, *NDVI*, population density index, distances to rivers, *MNDWI*, *TWI*, and lithology (Figure 4). 

It is significant to determine an appropriate spatial resolution of grid cells for predicting landslide susceptibility. This is because too low of a spatial resolution cannot ensure the reliability of the LSP while too high of a spatial resolution will strongly increase the complexity of the LSP modeling [71,72]. In this study, the original spatial resolutions of the grid cells of the DEM and Landsat TM images were both 30 m. This resolution value can effectively characterize the topography of Shicheng County and can avoid excessive computation [73]. In addition, a lot of literature suggests that a spatial resolution of 30 m is feasible and satisfactory for LSP [6,30,48,70,74,75]. Hence, the spatial resolution of grid cells in this study was set to 30 m. 

#### 3.1.2. Frequency Ratio Values of Environmental Factors

**Figure 5:**
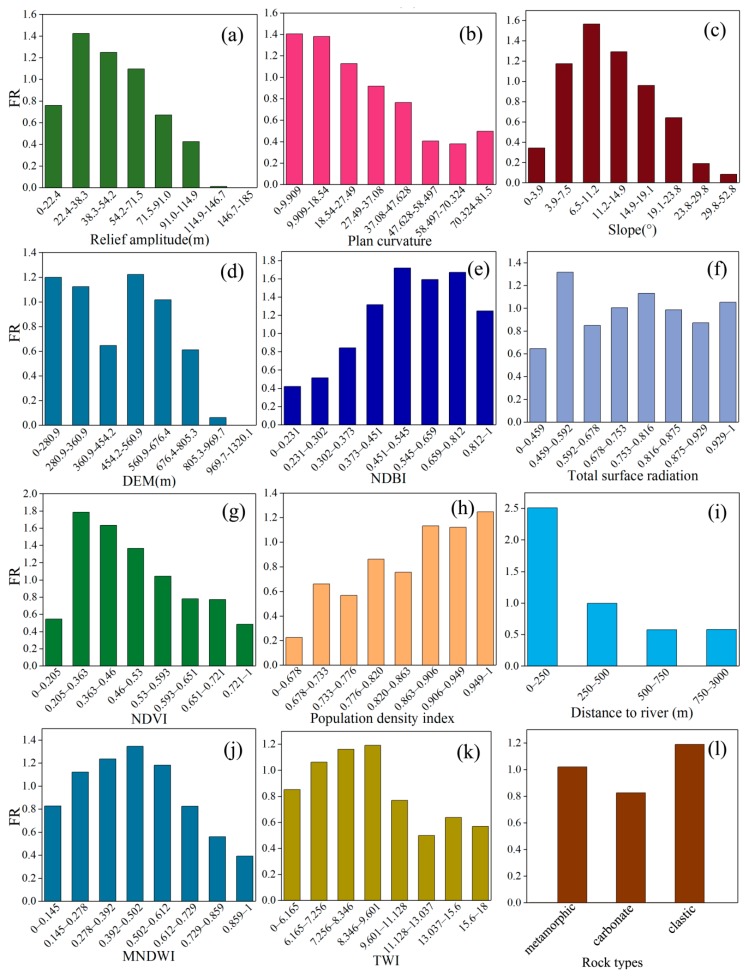
Frequency ratio values of environmental factors (aspect and profile curvature are not presented).

The frequency ratio values of these environmental factors were calculated to express the nonlinear correlations between environmental factors and landslides, as shown in Figure 5. The relief amplitude often appears in the range of 22–71. The plan curvature reflects the convergence and divergence of surface water flow [34], and the region of 0–27.5 is indicative of landslide events. The slope shows the difference in the surface water collecting capacity [76]. In addition, the aspect is closely related to landslide development and is often considered in the LSP, which is prone to landslide events in the ranges of 67.5–157.5 and 247.5–292.5. *NDBI* and population density intensively embody the range of human engineering activities with a high landslide probability. *NDVI* and total surface radiation reflect the impact of land cover on landslides [1], and an *NDVI* of 0.2–0.59 and a total surface radiation of 0.46–0.59 indicate a favorable environment for landslide occurrence. Moreover, the distances to rivers are the most common hydrologic environmental factor that affects the development of landslides, and the results show that the area with a distance to rivers within 250 m had the densest landslides. *MNDWI* was also selected as an environmental factor, and the landslides were mostly distributed in the range of 0.145–0.612. *TWI* reflects the centralized distribution state of water on the surface [77], and many landslides occur in range of 6.1–9.6. In addition, landslides are more likely to occur in the area with clastic rock than areas with metamorphic and carbonate rocks. 

#### 3.1.3. Model Training and Testing Datasets

It is necessary to prepare the training dataset and the test dataset for model training and testing prior to the prediction and modeling of landslide susceptibility. In this study, 369 landslides were converted into 2709 grid points in ARCGIS 10.2 software and randomly divided such that 70% and 30% went toward constructing the training and test sets, respectively. The same number of non-landslides were randomly divided using the same proportions to satisfy the binary classification condition. In addition, the *FR* values of the 14 environmental factors were used as input variables for the machine learning models, and the corresponding landslide and non-landslide results were marked as 1 and 0, which were treated as model output variables when constructing the dataset.

### 3.2. LSP Results of Shicheng County

The LSP of Shicheng County was implemented using each of the cascade-parallel LSTM-CRF, LR, C5.0 DT, and MLP models.

#### 3.2.1. LSP Using the Cascade-Parallel LSTM-CRF Model

**Figure 6:**
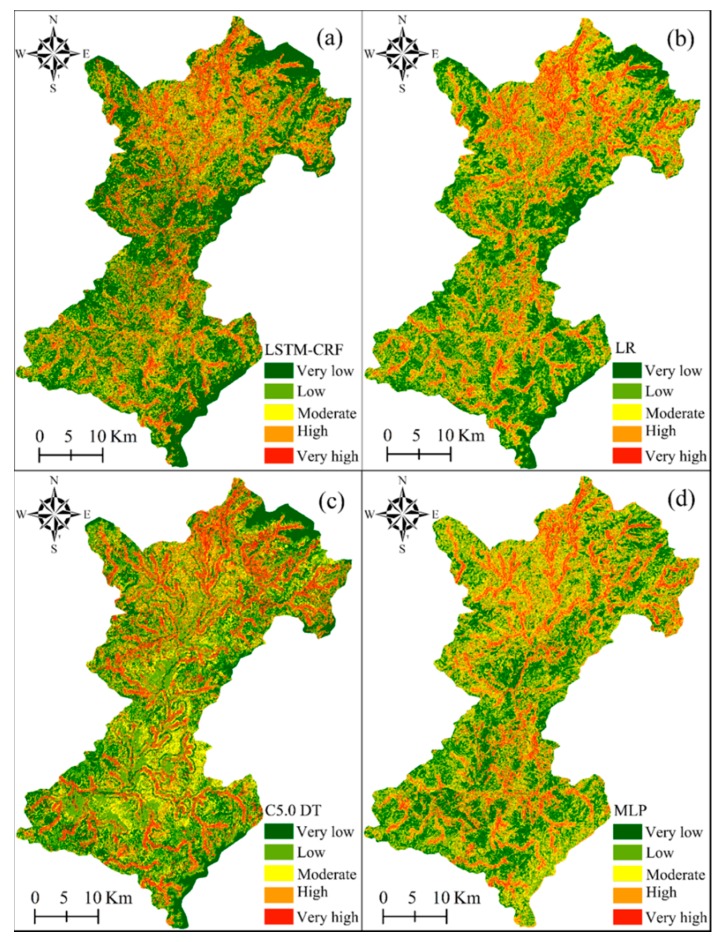
Landslide susceptibility map (LSM) produced by the four models. LR: Logistic regression, C5.0 DT: C5.0 decision tree, MLP: Multilayer perceptron.

**Figure 7:**
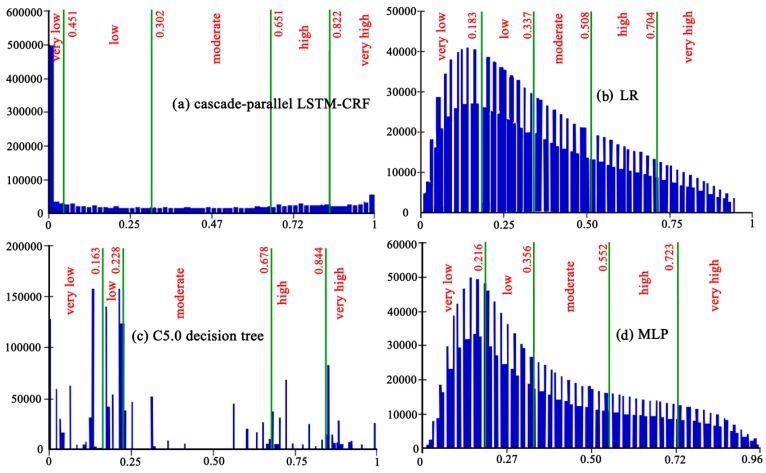
Landslide susceptibility index (LSI) distribution features of (**a**) LR, (**b**) MLP, (**c**) C5.0 decision tree, and (**d**) cascade-parallel LSTM-CRF.

The hardware configuration required for the cascade-parallel LSTM-CRF model in the operating environment is shown in Table 1. In this study, the hyper-parameter search was adopted to acquire the optimal parameters in the cascade-parallel LSTM-CRF, where the number of LSTM units, learning rate, and total training parameters were 5, 0.001, and 40,432, respectively. The trained cascade-parallel LSTM-CRF was used to predict the landslide susceptibility indices (LSIs) (Figure 7a) and to obtain the LSM (Figure 6a). In addition, the study area was divided into five categories, namely extremely low, low, moderate, high, and extremely high, using Jenks natural breaks optimization, and the corresponding area proportions were 33.56%, 25.41%, 18.33%, 10.98%, and 11.72%, respectively (Figure 7a). 

#### 3.2.2. LSP Using the LR Model

LR is a common statistical model that is widely used in LSP. The advantage of LR is that the independent variables need not be normally distributed, and the dependent variables can be continuous, discrete, and binary [78]. LR can define the relationship between the landslide occurrence and environmental factors and give the landslide coefficient of the corresponding factors. The logistic regression model can be expressed as follows:

(18) $$z=a_{0}+a_{1}x_{1}+a_{2}x_{2}+\cdots +a_{n}x_{n},$$

(19) $$P=ln(\frac{p}{1-p})=\frac{1}{1+e^{-z}},$$

where z is the dependent variable, including landslides (1) and non-landslides (0); $a_{0}$ is the regression intercept; $x_{n}\left(n=1,2,\cdots ,n\right)$ is the environmental factor; $a_{n}(n=1,2,\cdots ,n)$ is the regression coefficient; and P is the probability of landslide occurrence with a range from 0 to 1.

Considering the information redundancy between factors, 14 input variables were adopted to conduct correlation analysis in SPSS 23.0 software, and the results showed that the correlation between the factors was weak. All factors were imported into the LR model for calculation, and the profile curvature, relief amplitude, and total surface radiation were removed in the first calculation because the significance value was greater than 0.05. The remaining 11 environmental factors were imported into LR again, and the results are shown in Table 2. The regression coefficients of all factors were positive, indicating that each factor had a catalytic effect on the generation of landslides. Furthermore, lithology was the most influential factor for landslides in Shicheng County, followed by slope, *MNDWI*, plane curvature, population density, elevation, and *NDBI*. The regression coefficients of each factor were used to obtain the LSIs (Figure 7b) of the study area to generate the LSM (Figure 6b). The research area was divided into the five categories of extremely low, low, moderate, high, and extremely high, with the corresponding area proportions of 28.64%, 23.81%, 21.33%, 13.43%, and 12.79%, respectively (Figure 7b).

#### 3.2.3. LSP Using the C5.0 DT Model

The C5.0 DT is a tree structure that can intuitively interpret decision rules based on input conditions. The sample data were segmented using the input variable with the highest information gain in entropy, and the information gain reflects the change of variable information before and after the data are divided. The smaller the new conditional entropy change and the greater the information gain, the better the prediction accuracy of the model [79,80]. Moreover, the pruning of each leaf node is crucial to improving the model accuracy, which is beneficial for removing the environmental factors that have an insignificant effect on the decision results. The lower the purity of each leaf, the higher the classification accuracy. Additionally, the tree growth is limited with the pruning degree to avoid over-fitting of the model and to obtain a concise and accurate model.

In this study, the modeling of C5.0 DT was conducted in SPSS Modeler 18.0 software, the main contents of which included the input of variables, tree growth, tree pruning, and model validation. The boosting algorithm was adopted to improve the model prediction accuracy, and the pruning degree and the number of each branch node are the default values. The LSIs were calculated using the trained C5.0 DT, as shown in Figure 7c. The LSM generated in Shicheng County is shown in (Figure 6c). Furthermore, the study area was divided into extremely low, low, moderate, high, and extremely high areas, with the corresponding areas accounting for 30.38%, 24.13%, 19.74%, 12.19%, and 13.55%, respectively (Figure 7c).

#### 3.2.4. LSP Using the MLP Model

MLP is an artificial neural network technology that has been widely used in pattern recognition and classification [81], and it is often adopted for solving the complicated nonlinear problem between landslides and various environmental factors in the process of LSP. In addition, the MLP’s advantage is its powerful ability to work with inaccurate and fuzzy data [82], and the main components of MLP include an input layer, a hidden layer containing at least one neuron, and an output layer. Random initial weights are assigned among all neuron nodes, and the hidden layer does not contact the external environment, which addresses the sum of the product of input values and connection weights between each layer with a nonlinear function.

The neural network algorithm simulates human learning primarily by strengthening and adjusting the connection weight between the input layer and the output layer, and the performance in the network is used to update the internal system structure due to an external stimulus [83]. In this study, an extensively applied backpropagation algorithm was adopted to construct the MLP neural network. MLP neural network modeling consists of three stages: (1) in the process of the forward transmission of the input data, the sigmoid function is used to process the sum of the products of the weights between layers, and the error between the output value and the theoretical value is calculated; (2) in the iterative process of backward transmission, the calculated error value is used to continuously update the connection weights to obtain the optimal error value; and (3) the trained network structure is used in the classification and prediction of continuous data in the entire research area.

The number of required neurons in a single hidden layer was calculated to be 25 based on the minimum prediction error method. Therefore, the numbers of neurons in the input layer, the hidden layer, and the output layer were 14, 25, and 1, respectively, and via different tests, the momentum, learning rate, and training time were 0.36, 0.05, and 500, respectively. Moreover, the trained MLP model was used to calculate the LSIs (Figure 7d) and to acquire the LSM (Figure 6d), which was divided into the five categories of extremely low, low, moderate, high, and extremely high, with the corresponding area proportions of 27.7%, 22.9%, 22.7%, 16.9%, and 9.9%, respectively (Figure 7d).

### 3.3. Accuracy Evaluation

Quality evaluation of the models is a key step in successful landslide prediction. In this study, the predictive rate curves and statistical indicators were used to assess the predictive performance of the models.

#### 3.3.1. Predictive Rate Accuracy

The performance evaluation of the model is related to the model’s application of LSP in the study area [84]. To analyze and compare the prediction ability of each model, the prediction rate curve was adopted to evaluate the fitting degree between landslide grid cells in the testing dataset and the predicted landslide susceptibility indices in the study area. First of all, the calculated LSI values were sorted in descending order. Second, these sorted LSI values were categorized into 20 equal intervals with 5% cumulative intervals. Third, the percentage of the landslide grid cells in the testing dataset of each interval was determined based on the former obtained 20 equal intervals. At last, the prediction rate curves of all four models were drawn. The area under the predictive rate curve (AUC) could clearly explain the LSP performance of the model with the threshold value, which was closer to 1, indicating that the model’s performance was better. 

**Figure 8:**
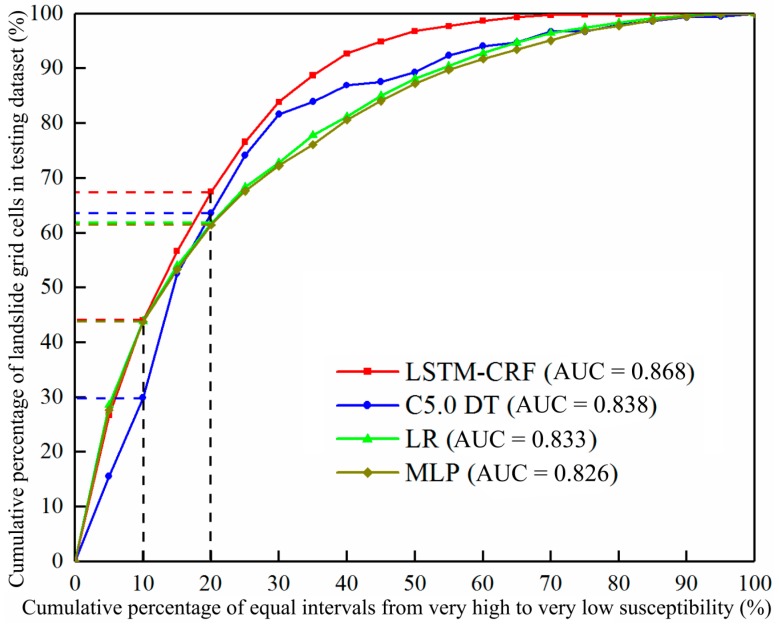
Prediction rate curves of LSIs calculated by the four models.

The predictive rate curves for different models are shown in Figure 8. In the top 10% of the very high susceptibility index interval, the cumulative percentages of landslide grids in the test set of the cascade-parallel LSTM-CRF, C5.0 DT, LR, and MLP models were 44.04%, 29.84%, 43.85%, and 43.81%, respectively. Moreover, in the top 20% of the study area with a high susceptibility index and above, the percentages of landslide grids in the corresponding models were 67.48%, 63.58%, 61.68%, and 61.43%, respectively. The prediction rate curve intuitively reflected the fact that the cascade-parallel LSTM-CRF, LR, and MLP models had a positive prediction performance in very highly prone areas. Overall, the AUC values of the cascade-parallel LSTM-CRF, C5.0 DT, LR, and MLP models were 0.868, 0.838, 0.833, and 0.826, respectively, indicating that the cascade-parallel LSTM-CRF model had a better predictive ability than other models.

#### 3.3.2. Statistical Index Accuracy

In this study, the statistical indexes, including the positive predictive rate (*PPR*), negative predictive rate (*NPR*), and total accuracy (*TA*), were used to measure the predictive performance of the models. The positive and negative predictive rates respectively represent the proportion of landslides and non-landslides that were correctly classified in the actual statistical process. Total accuracy represents the total predictive rate with which landslides and non-landslides are correctly classified. The corresponding expressions are given as:

(20) $$PPR=\frac{TP}{TP+FP}$$

(21) $$NPR=\frac{TN}{TN+FN}$$

(22) $$TA=\frac{TP+TN}{TP+TN+FP+FN}$$

where TP and FP respectively represent the number of landslide and non-landslide grids that are classified correctly, and TN and FN respectively represent the number of landslide and non-landslide grids that are classified mistakenly.

The performance of each model obtained using statistical indicators is presented in Table 3. In the predictive statistics process, the positive prediction rate of the C5.0 DT model produced the highest proportion of correct landslide classifications (76.78%), followed by the cascade-parallel LSTM-CRF model (72.44%), the MLP model (71.58%), and the LR model (71.04%). Regarding correct non-landslide classification, the cascade-parallel LSTM-CRF model produced the highest negative predictive rate of 80%, and those of the MLP, LR, and C5.0 DT models were 71.46%, 70.83%, and 69.73%, respectively. The cascade-parallel LSTM-CRF model has the best prediction performance regarding both landslides and non-landslides, with a total overall accuracy of 75.69%, followed by C5.0 DT (72.72%), MLP (71.61%), and LR (70.94%). In terms of the classification accuracy of landslides and non-landslides, the cascade-parallel LSTM-CRF model was more suitable than the other traditional models for LSPs.

## 4. Discussion

### 4.1. Discussion of the LSP Model Accuracy

The prediction rate and statistical indexes of the model accuracies evaluation show that the cascade-parallel LSTM-CRF model had the highest LSP performance, followed by the C5.0 DT, LR, and MLP models. However, it is not guaranteed that the deep learning model in this paper, among all machine learning models, is the optimal choice; however, the comparison of the models can reflect the ability of the proposed model to indeed overcome the shortcomings of traditional machine learning models. The cascade-parallel LSTM is one of the better performing models among all machine learning models. In further research, we aim to compare as many other machine learning models as possible to find the shortcomings of the proposed model and improve the model as much as possible to improve the accuracy of the landslide susceptibility predictions.

### 4.2. Model Iteration and Accuracy

**Figure 9:**
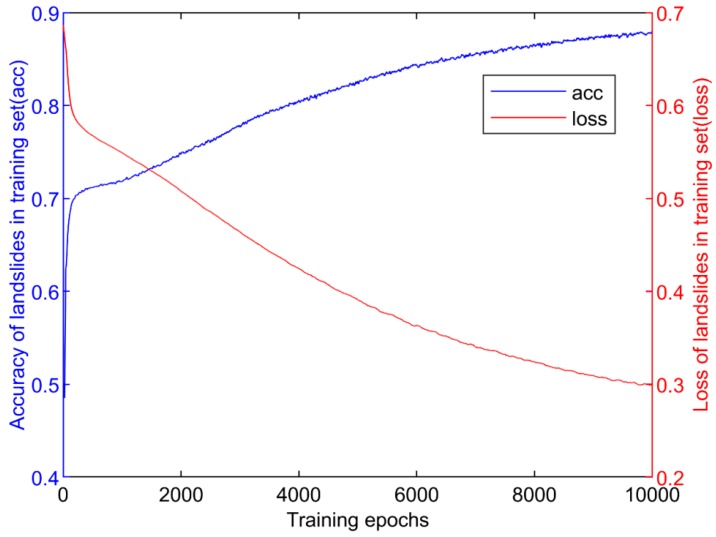
Loss and accuracy curves throughout the training of the models.

The loss is defined as the error between the theoretical value and the predicted value, which decreases with the updating of weights during backpropagation. The accuracy refers to the degree to which the predicted value is close to the theoretical value. The variation curves of the loss and accuracy in the model with a varying number of iterations are shown in Figure 9. The loss dropped sharply within 4000 iterations and stabilized at approximately 0.3 after 9000 iterations. During the same period, the accuracy of the model increased from 0.7 to 0.85, increased slowly until it became stable, and the final accuracy reached approximately 0.868.

### 4.3. Distribution Characteristics of Landslide Susceptibility

Landslide susceptibility maps obtained from the above four models are shown in Figure. According to the susceptibility index of the entire study area, the distribution of the susceptibility calculated by each model was roughly similar, and the distribution rules were consistent with the actual geological environment in the study area. According to superposition analysis of the landslide susceptibility maps and the environmental factor maps, it was observed that very high and high prone areas were mainly distributed near rivers, where *NDBI* and population density were relatively high, indicating that human engineering activities had a great impact on landslides. Moreover, most of the landslides were distributed in areas with a small *NDVI* and a large total surface radiation, which indicates that vegetation coverage had a certain inhibitory effect on landslide occurrence. Furthermore, landslides were always concentrated in brittle rock areas, such as areas with metamorphic and clastic rocks, which can create a favorable environment for the development of landslide events. 

In contrast, very low and low-landslide-prone areas were primarily distributed in areas far from rivers and carbonate rocks, which had relatively dense vegetation coverage, poor impounding conditions, less human activity, and a low probability of landslide occurrence. In addition, carbonate rocks were more conducive to slope stability and not conducive to landslides compared with metamorphic rocks and clastic rocks.

### 4.4. Advantages of the Cascade-Parallel LSTM-CRF Model 

A cascade-parallel LSTM-CRF model for landslide prediction based on deep learning was proposed in this study. Compared with other traditional prediction models, this model has the following advantages in LSP: (1) The prediction accuracy obtained by this algorithm was high, which was mainly embodied in the correct classification stage of landslides and non-landslides. The total predictive rate in the process of training was 75.67%, and the precision of statistical indicators in the test set was 0.868, both higher than those of the C5.0 DT, LR, and MLP models. (2) This algorithm is mainly based on deep learning and is data-driven, which overcomes the limitations of traditional prediction models related to input data, such as the need for substantial prior knowledge, the satisfaction of certain distribution characteristics, mutual independence between factors, etc. Hence, the algorithm greatly improves the applicability in landslide prediction and has a high practical value. (3) The main feature of this algorithm is the adoption of the cascade-parallel LSTM structure, which uses the perception range characteristics of different LSTM structures as data features and extracts and fuses the features of different input factors to acquire deeper and more complete features of each factor; furthermore, dropout is adopted to prevent over-fitting in the training process. (4) Considering that the susceptibility of a grid cell is affected by the susceptibility results of adjacent grids, this study introduced CRF, and the cross entropy function is used to change the direction of the gradient descent in the algorithm and accelerate the model convergence to optimize the model.

## 5. Conclusions

In this study, the cascade-parallel LSTM-CRF model was constructed by introducing a deep learning algorithm and combining it with CRF to predict landslide susceptibility in Shicheng County based on remote sensing images and GIS. The cascade-parallel LSTM-CRF model was compared with traditional prediction models, and 14 environmental factors were chosen based on the geological conditions relevant to landslide occurrence. The models were trained and tested based on adopting data samples, which were randomly split using a 70/30 training/testing ratio. Finally, the predictive rate curve and statistical indices were used to evaluate the performance of each model.

The results show that the cascade-parallel LSTM-CRF model had a better prediction performance than other models. The advantages of this algorithm are listed as follows: (1) The proposed algorithm has a higher LSP performance than traditional machine learning algorithms. (2) The proposed algorithm overcomes the limitations associated with traditional machine learning algorithms requiring substantial prior knowledge. Therefore, this algorithm is universal and practical, and can be applied to a wider range of landslide data predictions. (3) The stacked structure was adopted in this algorithm. Using the cascade-parallel LSTM, different layers of features can be extracted and merged from different layers, such as concrete and abstract. At the same time, the strategy of preventing over-fitting of the network was adopted, such that it can extract more comprehensive and accurate landslide features that facilitate classification. (4) CRF was further introduced in the cascade-parallel LSTM in this study. The predicted results are optimized by calculating the energy relationship between the two grid points, optimizing the extracted features and smoothing the predicted results of mutations. 

Moreover, the distribution results of landslide susceptibility of each model were consistent with the actual geological environment in the study area. In conclusion, the introduction of the deep learning algorithm is of great significance to the prediction of landslide susceptibility, which can supply the local government with theoretical guidance for the rational allocation of land resources and the implementation of disaster prevention and mitigation measures.

## Figures and Tables

**Table 1:** Software environment and hardware configuration in the experiment.

Software/Hardware	Parameters
Software Environment	TensorFlow1.4.0 + Keras2.1.4 (for cascade-parallel LSTM-CRF)
	SPSS Modeler 18.0 + Matlab 2018 (for logistic regression, multilayer perceptron, and C5.0 decision tree)
CPU	Intel(R) Core(TM) i5-7400@3.00 GHz
GPU	Nvidia GeForce GTX1080
RAM	8.00 GB DDR3
Hard Disk	Western Digital WDC WD10EZEX-08WN4A0

**Table 2:** The parameters in the LR model.

Environmental Factors	B	S.E.	Wald	Sig.	Exp (B)
Elevation	0.827	0.145	32.406	0	2.287
Slope	1.276	0.080	252.078	0	3.583
Aspect	0.691	0.159	18.917	0	1.996
Plan curvature	1.088	0.093	138.101	0	2.968
*MNDWI*	1.093	0.121	81.136	0	2.983
Distance to river	0.627	0.039	264.112	0	1.872
*TWI*	0.624	0.177	12.462	0	1.866
Lithology	2.397	0.269	79.468	0	10.990
*NDVI*	0.402	0.141	8.156	0.004	1.494
*NDBI*	0.810	0.102	62.701	0	2.249
Population density	0.899	0.150	36.063	0	2.457
Constant	−11.385	0.497	524.967	-	-

B is the regression coefficient, S.E. is the standard error, Wald is the Wald chi-square test, Sig. is the significance of the regression coefficient, and Exp(B) is the power of the regression coefficient.

**Table 3:** Comparison between Landslide prediction performances.

Prediction Performance	Prediction Models			
	Cascade-Parallel LSTM-CRF	C5.0 DT	LR	MLP
True positive	673	529	574	582
True negative	556	652	578	581
False positive	256	160	234	231
False negative	139	283	238	230
*PPR* (%)	72.44	76.78	71.04	71.58
*NPR* (%)	80.00	69.73	70.83	71.64
*TR* (%)	75.67	72.72	70.94	71.61
